# A meta-analysis of the effects of combined interventions centered on cognitive training on cognition, executive function, and negative emotions in patients with Alzheimer’s disease

**DOI:** 10.3389/fneur.2026.1883995

**Published:** 2026-09-15

**Authors:** Xingjun Zhou, Yuanye Hu, Zixuan Yang

**Affiliations:** Department of Leisure Services Sports, Pai Chai University, Daejeon, Republic of Korea

**Keywords:** Alzheimer’s disease, cognitive function, cognitive training, combined intervention, executive function, negative emotions, network meta-analysis

## Abstract

**Objective:**

To systematically compare the effects of five intervention strategies centered on Cognitive Training (CT), including Routine Care (RC), CT, Physical Exercise (PE), Cognitive Plus Exercise Training (CET), and Cognitive Plus Non-Invasive Brain Stimulation (CNIBS), on cognitive function, executive function, and negative emotions in patients with Alzheimer’s disease, using a network meta-analysis. The relative efficacy of different interventions was ranked to provide evidence-based support for optimizing combined treatment strategies in clinical practice.

**Methods:**

PubMed, Embase, Web of Science, Cochrane Library, and EBSCO databases were systematically searched to identify randomized controlled trials on CT and its combined interventions for Alzheimer’s disease published from database inception to April 2026. A network meta-analysis was conducted within a frequentist framework using Stata 17.0 software. The efficacy of interventions was ranked using the surface under the cumulative ranking curve.

**Results:**

A total of 19 randomized controlled trials involving more than 2,000 patients were included. The network meta-analysis showed that, for cognitive function, CNIBS (SUCRA = 75.4%) and CET (SUCRA = 72.4%) ranked first and second, respectively, and were significantly superior to RC and single interventions. For executive function, CET ranked highest (SUCRA = 99.9%) and was significantly superior to PE alone (SMD = 3.71, 95% CI: 3.16 to 4.26) and RC (SMD = 1.69, 95% CI: 0.48 to 2.91), other interventions do not offer a significant advantage over RC. For negative emotions, CET ranked highest (SUCRA = 83.7%), followed by PE alone (SUCRA = 57.9%), and CNIBS was also superior to CT alone (SMD = 1.07, 95% CI: 0.53 to 1.61).

**Conclusion:**

Combined interventions centered on CT are superior to single interventions in improving multidimensional symptoms in patients with Alzheimer’s disease. Among them, CET shows the most prominent advantages in improving executive function and negative emotions, whereas CNIBS shows the greatest effect on overall cognitive function. In clinical practice, individualized intervention strategies should be selected according to the primary rehabilitation goal.

## 1 Introduction

Alzheimer’s disease (AD) is a neurodegenerative disorder characterized by insidious onset and progressive deterioration. During disease progression, patients commonly develop memory decline, disorientation, and impairments in thinking and judgment, which may further involve broader higher-order brain functions (1–3). As the disease advances, cognitive impairment is often no longer limited to general cognitive performance, but may also extend to executive dysfunction and be accompanied by negative emotional symptoms such as depression, anxiety, and apathy, ultimately affecting patients’ ability to perform activities of daily living and maintain social functioning (4–7). These multidimensional impairments are interrelated, not only weakening patients’ capacity for independent living but also substantially increasing family caregiving pressure and the burden of long-term management (8–10). Therefore, how to simultaneously improve cognitive outcomes, maintain executive function, and alleviate negative emotions during disease management has become an important issue in comprehensive interventions for AD (11, 12).

At present, there is still no treatment capable of curing AD or reversing its disease course, and non-pharmacological interventions play an important role in long-term management (11–13). Routine Care (RC), Cognitive Training (CT), and Physical Exercise (PE) are commonly used interventions in clinical practice. CT helps activate and maintain cognitive processing and promotes training of attention, memory, and executive function (14, 15). PE may exert positive effects on cognition and emotion by improving cerebral blood flow, enhancing neuroplasticity, and improving overall functional status (16, 17). In recent years, non-invasive brain stimulation (NIBS), as an emerging neuromodulation approach, has also been increasingly applied in intervention studies involving patients with AD. Its potential mechanisms mainly involve modulation of cortical excitability and remodeling of neural networks (18, 19). Because AD involves multiple functional dimensions, including cognition, executive function, and emotion, single interventions often fail to comprehensively address patients’ complex therapeutic needs; therefore, combined interventions have gradually attracted increasing attention (19, 20). Among them, Cognitive Plus Exercise Training (CET) may generate synergistic effects through the complementary actions of behavioral training and physical activity, whereas Cognitive Plus Non-Invasive Brain Stimulation (CNIBS) may further enhance intervention effects through the additive effects of cognitive activation and neuromodulation (19–21). Different combined strategies vary in their mechanisms of action and intervention pathways, and their clinical benefits may also differ (18–21).

Although some randomized controlled trials have investigated the effects of CT, PE, NIBS, and their combined strategies in patients with AD, the existing evidence remains fragmented, and the comparative relationships among different studies are complex (14–21). On the one hand, the control conditions used across studies are inconsistent, involving RC, CT alone, PE alone, and other comparison pathways, making systematic evidence synthesis difficult (14, 16, 19). On the other hand, traditional meta-analyses are usually limited to pairwise comparisons and mainly answer whether a specific intervention is effective compared with a specific control, but they are less able to clarify the relative advantages and disadvantages among multiple single and combined therapeutic strategies (22, 23). For clinical practice, the more critical question is not merely “whether combined therapy is effective,” but “which combined strategy should be prioritized.” When direct head-to-head evidence is limited, network meta-analysis can integrate both direct and indirect evidence, simultaneously compare multiple interventions within a unified analytical framework, and rank their relative efficacy; therefore, it is more suitable for evaluating the comparative effects of different single and combined therapeutic strategies in patients with AD (22–24).

In addition, intervention effects may be influenced by dose-related factors, including training frequency, session duration, total intervention period, and overall intervention volume (17, 25). Even within the same intervention category, differences in dose arrangements across studies may lead to inconsistent treatment effects and become an important source of heterogeneity (17, 25). Therefore, in addition to comparing the relative effects of different intervention strategies, further dose analysis may help extend the question from “which strategy is more effective” to “under what dose conditions patients are more likely to benefit,” thereby providing supplementary evidence for developing more precise clinical intervention protocols (17, 23, 25).

Based on the above considerations, this study aimed to conduct a network meta-analysis to systematically compare the effects of RC, CT, PE, CET, and CNIBS on cognitive function, executive function, and negative emotions in patients with AD, and to rank the relative efficacy of different interventions. Meanwhile, dose analysis was conducted to further explore the relationships between dose-related factors, including training frequency, session duration, intervention period, and overall intervention volume, and intervention effects. This study is expected to provide evidence-based support for optimizing combined therapeutic strategies and formulating individualized intervention programs for patients with AD.

## 2 Methods

This study was designed and conducted in strict accordance with the Preferred Reporting Items for Systematic Reviews and Meta-Analyses (PRISMA) statement and the *Cochrane Handbook for Systematic Reviews of Interventions*. The protocol has been registered in the International Prospective Register of Systematic Reviews, with the registration number CRD420261390881.

### 2.1 Data sources and search strategy

Two researchers independently conducted the literature search. A search strategy combining subject headings and free-text terms was used to systematically search PubMed, Embase, Web of Science, Cochrane Library, and EBSCO databases. The search period ranged from January 2010 to April 2026. The language was restricted to English, and the study population was restricted to human participants. In addition, the reference lists of relevant reviews and included studies were manually searched to identify potentially eligible studies that may have been missed.

### 2.2 Study selection and inclusion criteria

The inclusion criteria were developed according to the PICOS framework as follows.

*P, Population:* Patients clinically diagnosed with AD, aged ≥18 years, with no restrictions on disease stage, disease subtype, or disease severity.

*I, Intervention:* The interventions included the following five types of rehabilitation interventions: RC, CT, PE, CET, and CNIBS.

*C, Comparison:* The control condition could include RC, sham intervention, wait-list control, or any other active rehabilitation therapy.

*O, Outcomes:* Studies were required to report at least one major outcome from the following dimensions:

1. Cognitive function: measured using validated comprehensive cognitive assessment tools, such as the Alzheimer’s Disease Assessment Scale–Cognitive Subscale (ADAS-Cog), Montreal Cognitive Assessment (MoCA), Mini-Mental State Examination (MMSE), or Addenbrooke’s Cognitive Examination III (ACE-III).
2. Executive function: assessed using specific executive function tasks or scales, such as the Trail Making Test (TMT, especially Part B), Verbal Fluency Test (VF), Digit Span Test (DST, especially backward span), Stroop Color-Word Test, Wisconsin Card Sorting Test (WCST), or Behavior Rating Inventory of Executive Function (BRIEF). For executive function, we included specific executive function tasks or scales, such as the Trail Making Test (TMT, especially Part B), Verbal Fluency Test (VF), Digit Span Test (DST, especially backward span), Stroop Color-Word Test, Wisconsin Card Sorting Test (WCST), or Behavior Rating Inventory of Executive Function (BRIEF). These instruments are well-validated neuropsychological tools that assess core components of executive function, including attentional control, inhibitory control, working memory, cognitive flexibility, and task switching. Although these tests differ in their specific emphasis, they collectively capture the executive function construct. They were selected because they were the most consistently reported executive function measures across the included studies, enabling sufficient statistical power for the network meta-analysis. We acknowledge that variability in these measures may contribute to between-study heterogeneity and have addressed this in the Discussion.
3. Negative emotions: measured using standardized emotional assessment scales, such as the Geriatric Depression Scale (GDS), Neuropsychiatric Inventory (NPI), Hamilton Depression Rating Scale (HAMD), Hamilton Anxiety Rating Scale (HAMA), Hospital Anxiety and Depression Scale (HADS), or Cornell Scale for Depression in Dementia (CSDD).

For cognitive function, we focused on overall cognitive performance as measured by validated comprehensive scales (e.g., ADAS-Cog, MoCA, MMSE, and ACE-III), which collectively cover multiple domains commonly affected in AD, including memory, language, and orientation. These global scales were selected as the primary outcome measures because they were consistently reported across the included studies, enabling sufficient statistical power for the network meta-analysis. We acknowledge that domain-specific neuropsychological tests may provide more granular information on specific cognitive functions; however, the heterogeneity in the use of such tests across studies precluded their inclusion in the current analysis.

*S, Study design*: Only randomized controlled trials were included.

The exclusion criteria were as follows: patients with severe systemic comorbidities; non-randomized controlled trials; animal experiments; reviews; conference abstracts; case reports; studies with data that could not be extracted; and studies for which complete data could not be obtained after contacting the authors.

### 2.3 Data extraction

Literature screening and data extraction were independently completed by two researchers. First, EndNote 20 software was used to remove duplicates from the search results. Subsequently, titles and abstracts were screened for initial eligibility, and the full texts of the remaining studies were reviewed to determine final inclusion. The two researchers cross-checked the screening results, and disagreements were resolved through discussion or consultation with a third researcher.

Data were extracted using a predesigned standardized form. Extracted information included basic study characteristics, including first author, publication year, and country; participant characteristics, including sample size in each group, age, and sex; intervention details, including specific therapy type, treatment frequency, session duration, and total intervention period; and outcome data, including pre- and post-treatment means, standard deviations, or change scores.

### 2.4 Risk-of-bias assessment of the included studies

Two researchers independently assessed the quality of the included studies using the Cochrane Risk of Bias tool version 5.1. The assessment covered the following seven domains: random sequence generation, allocation concealment, blinding of participants and intervention personnel, blinding of outcome assessors, incomplete outcome data, selective outcome reporting, and other sources of bias. Review Manager 5.3 software was then used to generate risk-of-bias plots. Each study was rated as “low risk,” “high risk,” or “unclear” in each domain. The two researchers cross-checked the assessment results, and disagreements were resolved through discussion or adjudication by a third researcher.

### 2.5 Operational definitions, classification rationale, and differences among the five rehabilitation interventions

This section aimed to clarify the operational definitions of each intervention category, explain the rationale for independent classification, and distinguish the core differences among intervention types. The classification was mainly based on the core mechanisms of action, training content, and consistency of each intervention in rehabilitation research, with the aim of preserving intervention-specific treatment effects and supporting individualized clinical decision-making. This was also the central objective of the comparative analysis in this study. Details are provided in Table 1.

**Table 1:** Operational definitions and classification rationale for the five rehabilitation interventions.

Intervention type	Operational definition	Classification rationale and differences
Routine Care (RC)	No structured rehabilitation training is provided. Only basic nursing care and routine medical care are delivered to maintain patients’ basic daily functioning.	Serves as the baseline control intervention, without any active rehabilitation training component, and differs from all rehabilitation programs that include specific training content.
Cognitive Training (CT)	Structured training aimed at improving cognitive function, including task-oriented exercises targeting cognitive domains such as attention, memory, and executive function.	The core mechanism is cognitive neuroplasticity. The intervention content focuses on cognitive processing and differs from PE interventions centered on physical activity.
Physical Exercise (PE)	Structured physical activity aimed at improving physical function, including aerobic exercise, resistance training, balance training, flexibility exercises, and related modalities.	The core mechanisms include peripheral physiological adaptation, such as cardiopulmonary function and muscle strength, and neuromuscular relearning. PE differs from CT, which is centered on cognitive processing.
Cognitive Plus Exercise Training (CET)	The cognitive–exercise rehabilitation model focused on in this study, including both simultaneous and sequential integration. This model requires patients to perform additional cognitive tasks on the basis of exercise training, thereby forming dual-task or multitask training.	The core feature is cognitive–exercise task integration, emphasizing the interaction between the two components. It differs from single-component CT or PE.
Cognitive Plus Non-Invasive Brain Stimulation (CNIBS)	Non-invasive brain stimulation techniques, such as transcranial direct current stimulation (tDCS) or transcranial magnetic stimulation (TMS), are combined with CT to modulate cortical excitability in target brain regions.	The core mechanism is the synergistic effect of CT plus neuromodulation. Exogenous modulation is used to enhance the effects of CT, distinguishing CNIBS from CT without neuromodulation.

### 2.6 Statistical analysis

Network meta-analysis was performed within a frequentist framework using STATA 17.0 software and its network package. All analyses were conducted using a random-effects model to account for heterogeneity across studies. For continuous outcomes, effect sizes were expressed as standardized mean differences (SMDs) with 95% confidence intervals (CIs), and Hedges’ g was used for small-sample correction to improve estimation precision. The SMD was chosen as the effect measure because the included studies used different validated instruments to assess the same underlying constructs. According to the Cochrane Handbook for Systematic Reviews of Interventions, SMD is the recommended metric when studies have used different measurement instruments to assess the same outcome domain and the instruments are not directly comparable. For cognitive function, although the included studies utilized MMSE, ADAS-Cog, MoCA, and ACE-III with distinct scoring ranges, all these instruments are validated measures of global cognitive function in older adults with cognitive impairment. Similarly, for executive function, the various instruments (TMT-B, Stroop, Digit Span, WCST) all assess core domains of executive function, and for negative emotions, the scales (GDS, NPI, HAMD, HADS) all measure negative affective symptoms. Pooling these measures via SMD is therefore methodologically justified.

For the assessment of network consistency, both global and local approaches were employed. Global inconsistency was evaluated using the design-by-treatment interaction model, which tests for the presence of any inconsistency across the entire network. Local consistency was assessed using the node-splitting method, which compares direct and indirect evidence for each closed loop in the network. A non-significant global test (*p* > 0.05) and non-significant node-splitting results (*p* > 0.05) were considered indicative of acceptable network consistency.

The surface under the cumulative ranking curve (SUCRA) was used to rank the efficacy of each intervention. This indicator is expressed as a percentage ranging from 0 to 100%, providing an intuitive estimate of the probability that each intervention is the optimal option. In the network plots, node size was proportional to sample size, and line thickness corresponded to the number of studies directly comparing two interventions. To assess the robustness of the evidence, comparison-adjusted funnel plots were generated to identify publication bias and small-study effects.

To explore potential sources of heterogeneity, we performed exploratory subgroup analyses stratified by intervention duration (categorized as ≤4 weeks, 5–8 weeks, 9–16 weeks, and >16 weeks) and weekly training frequency (categorized as ≤2, 3, 4, and 5 sessions per week). These cutoffs were chosen based on the distribution of the included studies. Subgroup differences were tested using the Q-test for heterogeneity between subgroups, and *I*^2^ statistics were calculated to assess within-subgroup heterogeneity. Of note, subgroup analyses stratified by specific measurement tools were not performed due to the limited number of studies, which would have resulted in extremely small subgroups with insufficient statistical power to yield meaningful comparisons. Instead, we have acknowledged the heterogeneity arising from the diversity of outcome measures in the Discussion section.

To assess the robustness of the findings, we conducted a sensitivity analysis by excluding studies rated as having a high risk of bias based on the Cochrane RoB 2.0 assessment. The network meta-analysis was re-run after excluding these studies, and the SUCRA rankings were compared with those from the main analysis to determine whether the exclusion of low-quality studies substantially altered the results. Due to the limited number of included studies, formal meta-regression and additional sensitivity analyses (e.g., excluding small-sample trials or using alternative effect size models) were not performed.

## 3 Results

### 3.1 Literature screening process

The literature screening process of this study is shown in Figure 1. The specific steps were as follows. First, a total of 4,025 relevant records were retrieved through the initial search. Subsequently, 273 duplicate records were removed using the automatic duplicate removal function of EndNote software combined with manual verification. The remaining 3,752 records were screened by two researchers based on titles and abstracts, after which 3,571 clearly irrelevant records were excluded. Finally, the full texts of 181 articles were retrieved for detailed review. After strict assessment against the inclusion criteria, 19 randomized controlled trials met the eligibility criteria and were included in this network meta-analysis.

**Figure 1:**
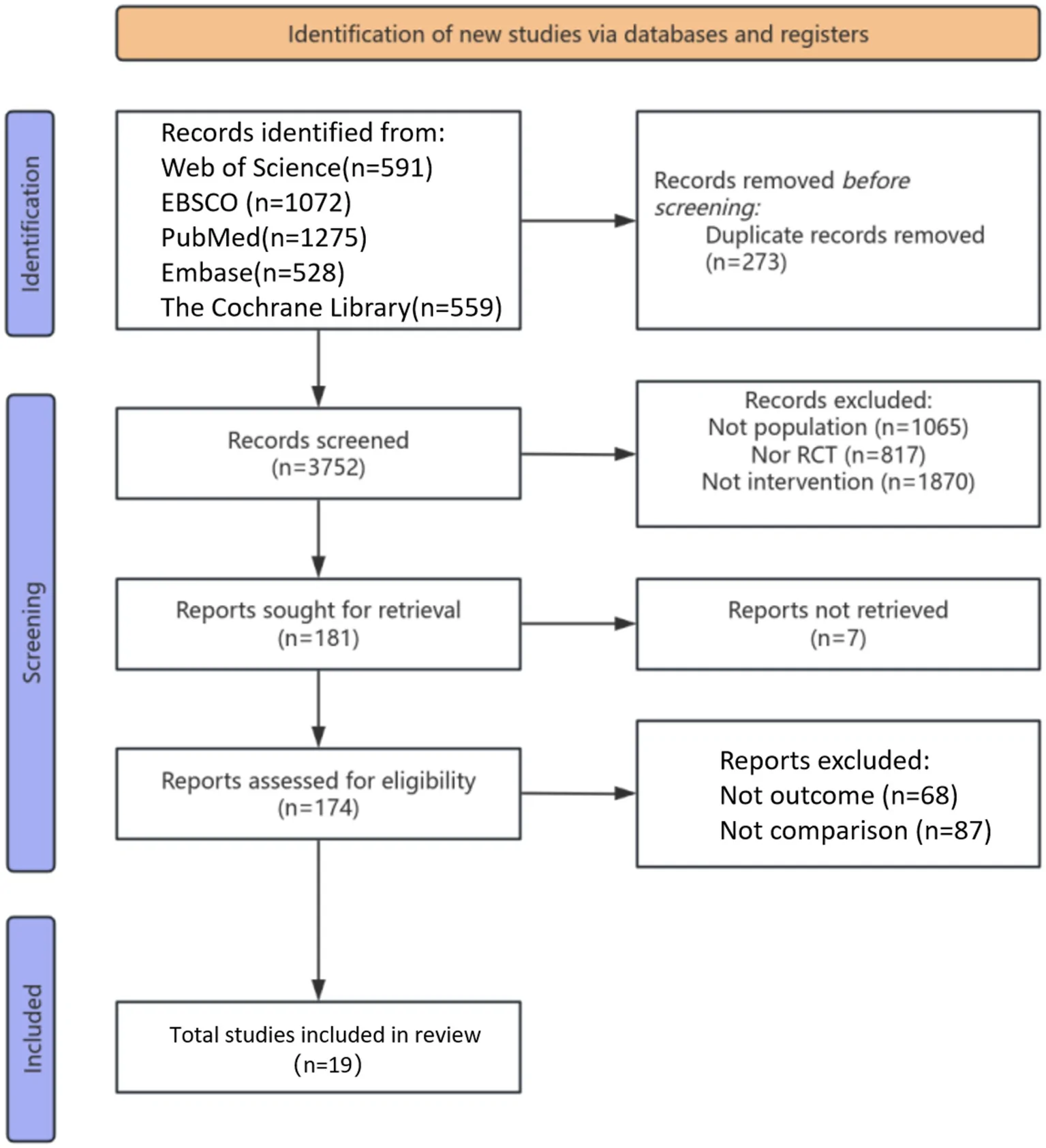
Flow diagram of the literature search process.

### 3.2 Quality assessment of the included studies

The certainty of the network meta-analysis results was assessed using the CINeMA framework. For cognitive function, the certainty of evidence across comparisons ranged from low to moderate. Specifically, the overall certainty was moderate for CET versus PE, CET versus RC, CT versus RC, and PE versus RC, whereas the certainty was low for CNIBS versus CT and CNIBS versus RC. The downgrading of evidence for cognitive function was mainly attributable to within-study bias, imprecision, heterogeneity, and publication bias. The significant Egger’s test (bias = 4.79, *p* < 0.001) indicated the presence of small-study effects or publication bias for this outcome, which particularly affected the evidence certainty for comparisons involving smaller studies (e.g., CNIBS vs. CT and CNIBS vs. RC).

For executive function, the certainty was moderate for CET versus PE, CET versus RC, and CNIBS versus PE, whereas the certainty was low for CNIBS versus CT. The non-significant Egger’s test (bias = 0.11, *p* = 0.986) indicated no strong evidence of publication bias for this outcome, and thus no additional downgrading was applied for this domain. Downgrading for executive function was mainly attributable to within-study bias, imprecision, and heterogeneity.

For negative emotions, the certainty of evidence was moderate for all comparisons, including CET versus PE, CET versus RC, CNIBS versus CT, CNIBS versus RC, and PE versus RC. Although the Egger’s test was significant (bias = 7.84, *p* = 0.008), suggesting potential publication bias for this outcome, the overall certainty remained moderate after considering the magnitude of the effect estimates and the consistency across studies. Downgrading for negative emotions was mainly attributable to within-study bias, imprecision, and heterogeneity, with publication bias also contributing to the downgrading.

Overall, the certainty of evidence ranged from low to moderate across all outcomes. Although some comparisons included a small number of studies, no obvious inconsistency was observed between direct and indirect evidence. Detailed CINeMA assessment results are shown in Figure 2.

**Figure 2:**
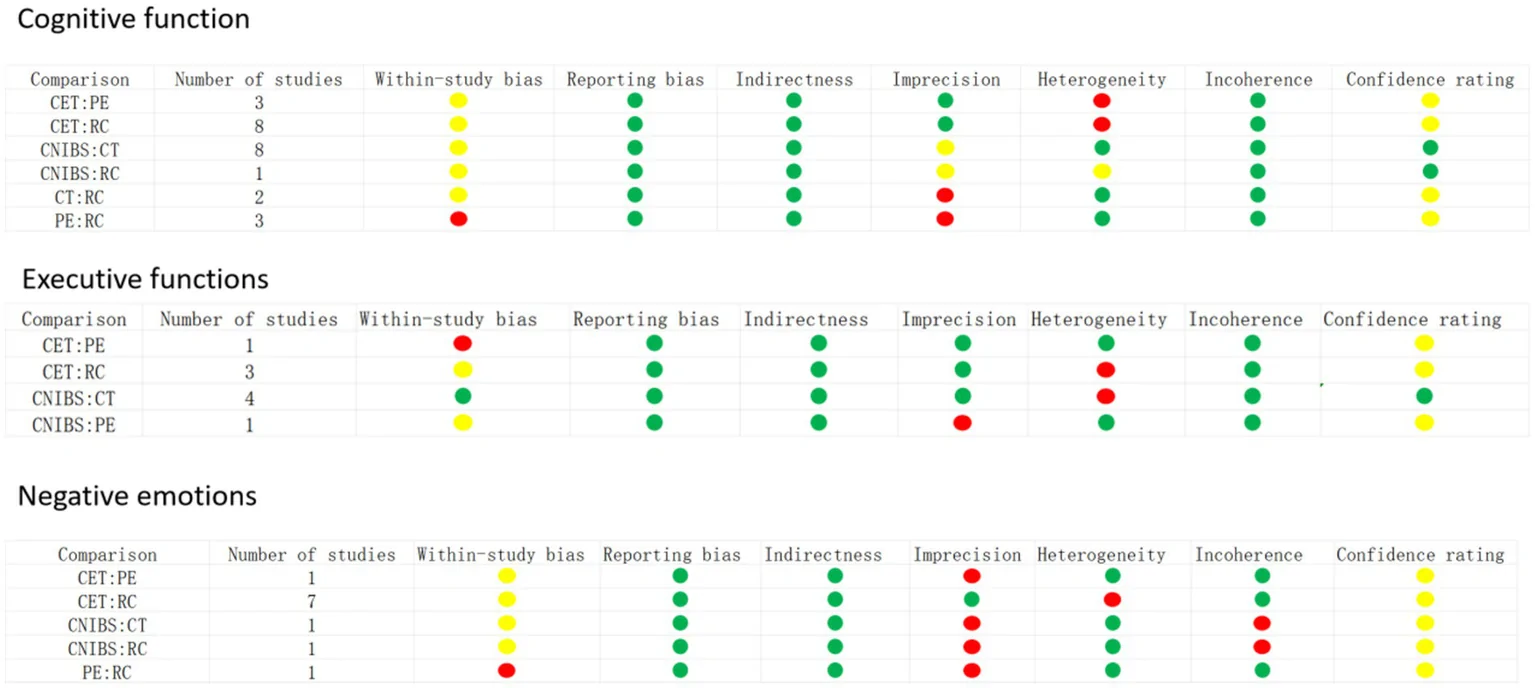
CINeMA quality assessment of the included studies.

### 3.3 Basic characteristics of the included studies

The basic characteristics of the 19 included studies are presented in Appendix 1. These studies were published between 2012 and 2025 and were conducted in multiple countries or regions worldwide, including Brazil, Italy, China, Spain, South Korea, Germany, Japan, Türkiye, Tunisia, Iran, the Czech Republic, and Finland. More than 2,000 patients with AD were included in total. In terms of interventions, the network covered the five rehabilitation types predefined in this study: RC, CT, PE, CET, and CNIBS. Among them, CET and CNIBS were the most frequently investigated intervention areas. The treatment period ranged from 2 weeks to 52 weeks, approximately 12 months, and the session duration ranged from 15 min to 2 h. The intervention frequency was mostly 2–5 sessions per week.

### 3.4 Risk-of-bias assessment

According to the Cochrane RoB 2.0 tool, the included studies generally showed good methodological quality in random sequence generation, with the vast majority rated as low risk. For allocation concealment and blinding of outcome assessors, approximately half of the studies provided sufficient methodological descriptions and met the criteria for low risk, whereas the remaining studies were rated as having unclear risk because of insufficient reporting. Due to the nature of rehabilitation interventions, most studies had difficulty implementing blinding of patients and therapists, resulting in generally high risk of bias in this domain. In terms of data integrity, incomplete outcome data and selective outcome reporting were rated as low risk in most studies. Notably, 6 studies were rated as high risk, and the remaining 13 studies were rated as low risk. Overall, the risk of bias in the included studies was acceptable and was predominantly low. Details are provided in Table 2, and the complete assessment plots are shown in Appendix 2.

**Table 2:** Risk of bias assessment of the included studies.

Included study	Randomization	Allocation concealment	Blinding - participants	Blinding - outcome assessment	Incomplete outcome data	Selective reporting	Other bias
Andrade, 2022	L	L	L	L	L	L	L
Cezar, 2021	L	L	L	L	L	L	L
Cezar, 2021 March	L	L	L	L	L	L	L
Chéour, 2023	L	U	H	L	L	L	L
Cotelli, 2014	L	U	L	L	L	L	L
Dengiz, 2024	L	U	L	L	L	L	L
Fang, 2025	L	L	L	L	L	L	L
Fonte, 2024	L	U	L	L	L	L	L
Koc, 2024	U	U	H	L	L	L	L
Kwon, 2025	L	L	H	L	L	L	L
Lee, 2016	L	U	L	L	L	L	L
Lu, 2019	L	L	L	L	L	L	L
Maci, 2012	L	H	L	L	L	L	L
Menengiç, 2022	L	U	H	L	L	L	L
Ohman, 2016	L	H	H	L	L	L	L
Shokri, 2024	L	U	L	L	L	L	L
Vecchio, 2022	L	U	L	L	L	L	L
Vostry, 2025	L	L	L	L	L	L	L
Zhang, 2019	L	U	L	L	L	L	L

### 3.5 Direct pairwise meta-analysis

The forest plots of the direct pairwise meta-analysis are shown in Appendix 3.

Compared with RC, CET showed a significant advantage in improving cognitive function (SMD = 1.33, 95% CI [0.42, 2.23], *p* = 0.004), although substantial heterogeneity was observed across studies (*I*^2^ = 94%), suggesting that the effect varied considerably among studies. PE did not show a significant effect compared with RC (SMD = 0.33, 95% CI [−0.58, 1.25], *p* = 0.47), with high heterogeneity (*I*^2^ = 87%). CT also did not reach statistical significance compared with RC (SMD = 3.65, 95% CI [−3.22, 10.52], *p* = 0.30), with extremely high heterogeneity (*I*^2^ = 94%). CNIBS showed no significant difference compared with RC (SMD = −0.08, 95% CI [−0.92, 0.75], *p* = 0.85). Among direct comparisons between active interventions, CET showed a significant cognitive improvement compared with PE alone (SMD = 1.67, 95% CI [0.34, 3.01], *p* = 0.01), although heterogeneity was high (*I*^2^ = 80%). CNIBS also showed a significant advantage over CT alone (SMD = 0.35, 95% CI [0.01, 0.68], *p* = 0.04), with moderate heterogeneity (*I*^2^ = 58%).

Compared with RC, CET showed a significant advantage in improving executive function (SMD = 1.69, 95% CI [0.48, 2.91], *p* = 0.006), although substantial heterogeneity was observed across studies (*I*^2^ = 93%), indicating considerable variation in effect estimates among studies. Among direct comparisons between active interventions, CET showed an extremely significant improvement in executive function compared with PE alone (SMD = 3.71, 95% CI [3.16, 4.26], *p* < 0.00001). CNIBS also showed a significant advantage over CT alone (SMD = 0.64, 95% CI [0.05, 1.22], *p* = 0.03), but heterogeneity was high (*I*^2^ = 71%). In addition, CNIBS did not reach statistical significance compared with PE alone (SMD = 0.54, 95% CI [−0.30, 1.38], *p* = 0.21).

Compared with RC, CET showed a highly significant effect in improving negative emotions (SMD = 2.70, 95% CI [1.31, 4.10], *p* = 0.0001), although substantial heterogeneity was observed across studies (*I*^2^ = 94%), indicating considerable variation in effects across studies. PE did not show a significant difference compared with RC (SMD = 0.31, 95% CI [−0.31, 0.93], *p* = 0.33). CNIBS also did not reach statistical significance compared with RC (SMD = 0.10, 95% CI [−0.73, 0.94], *p* = 0.81). Among direct comparisons between active interventions, CNIBS showed a significant advantage over CT alone (SMD = 1.07, 95% CI [0.53, 1.61], *p* = 0.0001). CET did not show a significant difference compared with PE alone (SMD = 0.33, 95% CI [−0.32, 0.97], *p* = 0.33).

### 3.6 Network meta-analysis

#### 3.6.1 Network plots of the included studies

The five nodes in the network plots represent the five intervention measures, and the lines between nodes indicate the presence of direct comparisons between interventions. Line thickness represents the number of studies directly comparing two interventions. Among the interventions, CET was the most extensively studied, whereas studies involving PE were relatively limited. The network plots of the outcome measures are shown in Figure 3.

**Figure 3:**
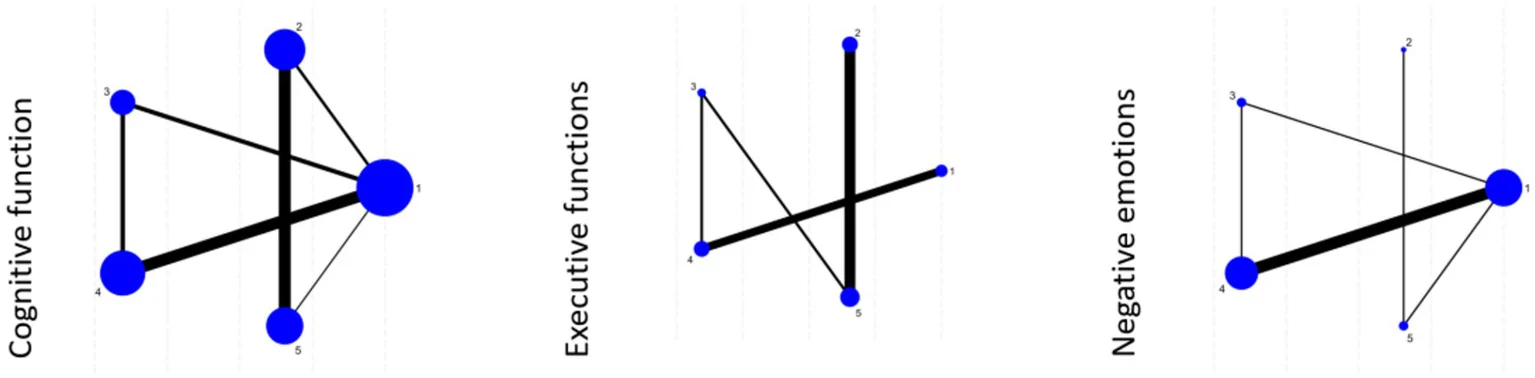
Network diagrams of evidence for each outcome. 1 = RC; 2 = CT; 3 = PE; 4 = CET; 5 = CNIBS.

#### 3.6.2 Ranking of different rehabilitation interventions

The ranking of different rehabilitation interventions is shown in Table 3 and Figure 4.

**Table 3:** Ranking of different rehabilitation interventions for improving outcomes.

Intervention	Cognitive function		Executive function		Negative emotions	
	SUCRA (%)	Rank	SUCRA (%)	Rank	SUCRA (%)	Rank
RC	16.2	5	70.4	2	33.1	4
CT	66.4	3	13.4	5	31.9	5
PE	19.7	4	20.5	4	57.9	2
CET	72.4	2	99.9	1	83.7	1
CNIBS	75.4	1	45.9	3	43.4	3

**Figure 4:**
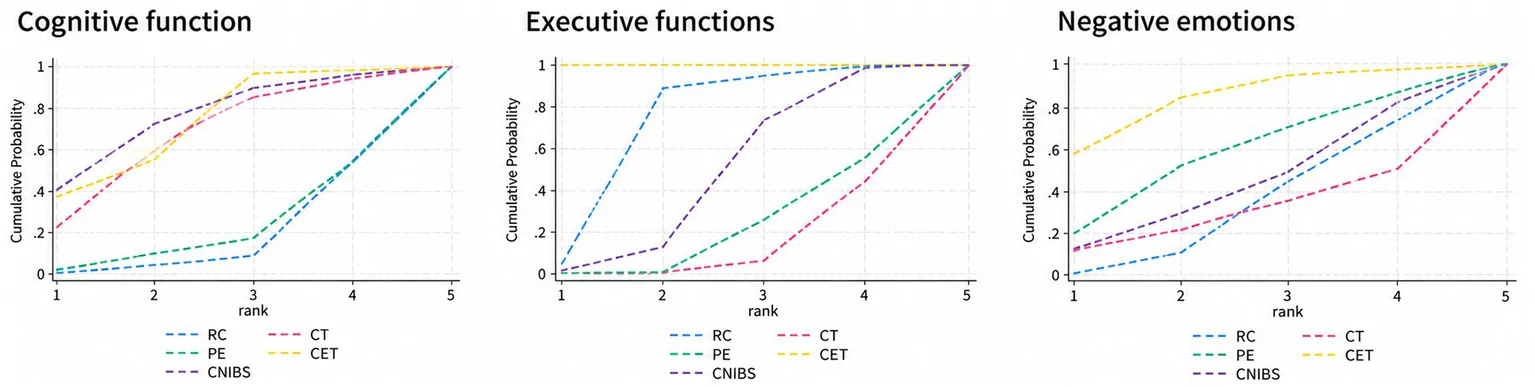
Cumulative ranking probability (SUCRA) curves for cognitive function.

For *cognitive function*, the ranking of the five rehabilitation intervention modes was as follows: CNIBS (SUCRA = 75.4) > CET (SUCRA = 72.4) > CT (SUCRA = 66.4) > PE (SUCRA = 19.7) > RC (SUCRA = 16.2). CNIBS had the highest probability of being the optimal intervention, CET and CT ranked second and third, respectively, and RC showed the weakest effect.

For *executive function*, the ranking of the five rehabilitation intervention modes was as follows: CET (SUCRA = 99.9) > RC (SUCRA = 70.4) > CNIBS (SUCRA = 45.9) > PE (SUCRA = 20.5) > CT (SUCRA = 13.4). CET had an extremely high probability of being the optimal intervention, with a SUCRA value approaching 100%. RC unexpectedly ranked second, whereas CT showed the weakest effect.

For *negative emotions*, the ranking of the five rehabilitation intervention modes was as follows: CET (SUCRA = 83.7) > PE (SUCRA = 57.9) > CNIBS (SUCRA = 43.4) > RC (SUCRA = 33.1) > CT (SUCRA = 31.9). CET had the highest probability of being the optimal intervention, PE ranked second, and CT showed the weakest effect.

It is important to note that the SUCRA rankings for cognitive function and negative emotions should be interpreted with particular caution. The significant Egger’s tests (cognitive function: bias = 4.79, *p* < 0.001; negative emotions: bias = 7.84, *p* = 0.008) indicate that small-study effects or publication bias may have inflated the effect estimates for these outcomes, potentially overestimating the relative advantage of interventions with smaller sample sizes. In contrast, the non-significant Egger’s test for executive function (bias = 0.11, *p* = 0.986) suggests that the rankings for this outcome are less likely to be biased by publication or small-study effects. These findings have been incorporated into the CINeMA evidence certainty assessment, and the evidence for cognitive function and negative emotions has been downgraded accordingly (Section 3.2). Therefore, the cognitive function and negative emotions SUCRA rankings should be interpreted with greater caution than the rankings for executive function.

#### 3.6.3 Pooled effect estimates for the primary outcomes

Compared with RC, CET showed a significant effect in improving cognitive function (SMD = 1.33, 95% CI [0.42, 2.23]), and CNIBS also showed a significant advantage over CT alone (SMD = 0.35, 95% CI [0.01, 0.68]). The ranking of different rehabilitation intervention modes for improving cognitive function was as follows: CNIBS (SUCRA = 75.4) > CET (SUCRA = 72.4) > CT (SUCRA = 66.4) > PE (SUCRA = 19.7) > RC (SUCRA = 16.2).

Compared with RC, CET showed a significant effect in improving executive function (SMD = 1.69, 95% CI [0.48, 2.91]). Compared with PE alone, CET also showed an extremely significant effect (SMD = 3.71, 95% CI [3.16, 4.26]). In addition, CNIBS showed a significant advantage over CT alone (SMD = 0.64, 95% CI [0.05, 1.22]). The ranking of different rehabilitation intervention modes for improving executive function was as follows: CET (SUCRA = 99.9) > RC (SUCRA = 70.4) > CNIBS (SUCRA = 45.9) > PE (SUCRA = 20.5) > CT (SUCRA = 13.4).

Compared with RC, CET showed a highly significant effect in improving negative emotions (SMD = 2.70, 95% CI [1.31, 4.10]). CNIBS also showed a significant advantage over CT alone (SMD = 1.07, 95% CI [0.53, 1.61]). The ranking of different rehabilitation intervention modes for improving negative emotions was as follows: CET (SUCRA = 83.7) > PE (SUCRA = 57.9) > CNIBS (SUCRA = 43.4) > RC (SUCRA = 33.1) > CT (SUCRA = 31.9).

#### 3.6.4 Consistency assessment

To evaluate the consistency of the evidence network, we performed both global and local inconsistency tests. The design-by-treatment interaction model yielded a non-significant global inconsistency test for all three outcomes (cognitive function: χ^2^ = 2.13, df = 2, *p* = 0.3439; executive function: χ^2^ = 0.45, df = 1, *p* = 0.5031; negative emotions: χ^2^ = 0.26, df = 1, *p* = 0.6113), indicating no statistically significant inconsistency across the entire network.

For local consistency, the node-splitting method was applied to each closed loop. The results showed that all direct and indirect evidence were consistent (all *p* > 0.05), with no significant disagreement detected for any comparison node across the three outcomes. These findings support the consistency assumption of the network meta-analysis and confirm the stability of the evidence network (Table 4).

**Table 4:** League table of pairwise comparisons among different rehabilitation interventions.

				
Cognitive function				
RC	−0.18 (−2.68, 2.32)	−1.69 (−4.40, 1.03)	−1.72 (−3.98, 0.54)	−0.19 (−1.34, 0.95)
0.18 (−2.32, 2.68)	PE	−1.50 (−3.03, 0.03)	−1.53 (−2.64, -0.43)	−0.01 (−2.46, 2.44)
1.69 (−1.03, 4.40)	1.50 (−0.03, 3.03)	CT	−0.03 (−1.53, 1.47)	1.49 (−1.18, 4.17)
1.72 (−0.54, 3.98)	1.53 (0.43, 2.64)	0.03 (−1.47, 1.53)	CNIBS	1.52 (−0.68, 3.73)
0.19 (−0.95, 1.34)	0.01 (−2.44, 2.46)	−1.49 (−4.17, 1.18)	−1.52 (−3.73, 0.68)	CET
Executive function				
RC	3.85 (1.56, 6.15)	0.69 (−0.09, 1.47)	0.15 (−1.63, 1.92)	2.14 (−0.32, 4.59)
−3.85 (−6.15, −1.56)	PE	−3.16 (−5.32, −1.00)	−3.71 (−5.17, −2.24)	−1.72 (−2.59, −0.84)
−0.69 (−1.47, 0.09)	3.16 (1.00, 5.32)	CT	−0.54 (−2.14, 1.05)	1.44 (−0.89, 3.77)
−0.15 (−1.92, 1.63)	3.71 (2.24, 5.17)	0.54 (−1.05, 2.14)	CNIBS	1.99 (0.28, 3.69)
−2.14 (−4.59, 0.32)	1.72 (0.84, 2.59)	−1.44 (−3.77, 0.89)	−1.99 (−3.69, −0.28)	CET
Negative emotions				
RC	−3.67 (−11.76, 4.42)	−2.60 (−8.53, 3.32)	−1.37 (−5.42, 2.69)	−2.71 (−4.77, −0.64)
3.67 (−4.42, 11.76)	PE	1.07 (−4.46, 6.60)	2.31 (−6.50, 11.12)	0.96 (−6.86, 8.78)
2.60 (−3.32, 8.53)	−1.07 (−6.60, 4.46)	CT	1.24 (−5.64, 8.11)	−0.11 (−5.66, 5.45)
1.37 (−2.69, 5.42)	−2.31 (−11.12, 6.50)	−1.24 (−8.11, 5.64)	CNIBS	−1.34 (−5.40, 2.71)
2.71 (0.64, 4.77)	−0.96 (−8.78, 6.86)	0.11 (−5.45, 5.66)	1.34 (−2.71, 5.40)	CET

Data are presented as standardized mean differences (SMD) with 95% confidence intervals in parentheses. SMD values in the upper triangle represent comparisons of the column-defining intervention versus the row-defining intervention; values in the lower triangle represent the inverse comparisons (negative SMD favors the column-defining intervention). Statistically significant comparisons (*p* < 0.05) are reported in detail in Section 3.6.3.

### 3.7 Assessment of publication bias

To evaluate the presence of publication bias and small-study effects, comparison-adjusted funnel plots were generated for each outcome (Figure 5). To further quantify the potential for publication bias, we performed Egger’s regression tests for each outcome. The results revealed that Egger’s test was significant for cognitive function (bias = 4.79, *p* < 0.001) and negative emotions (bias = 7.84, *p* = 0.008), indicating the presence of small-study effects or publication bias for these two outcomes. In contrast, the Egger’s test for executive function was not statistically significant (bias = 0.11, *p* = 0.986), suggesting no strong evidence of publication bias or small-study effects for this outcome. The significant results for cognitive function and negative emotions should be considered when interpreting the findings, and the corresponding funnel plots did show some asymmetry.

**Figure 5:**
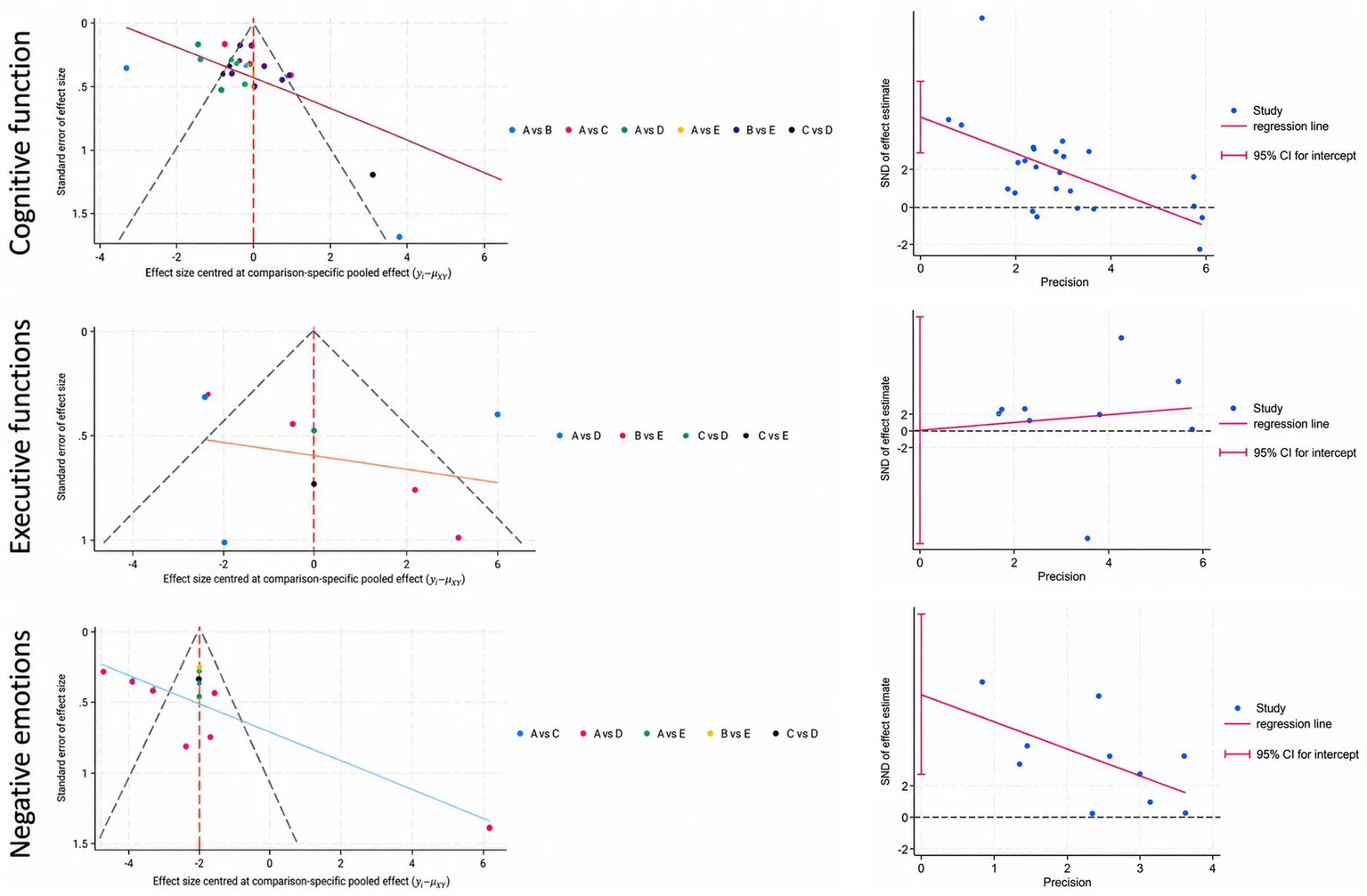
Comparison-adjusted funnel plots and Egger’s regression tests for publication bias. A = RC; B=CT; C=PE; D=CET; E = CNIBS.

Overall, these results indicate that publication bias cannot be completely ruled out for cognitive function and negative emotions, whereas the evidence for executive function appears more robust in this regard.

### 3.8 Subgroup analyses exploring sources of heterogeneity

To explore the potential sources of the substantial heterogeneity observed across the primary outcomes (*I*^2^ ranging from 87 to 94%), we conducted exploratory subgroup analyses stratified by intervention duration and weekly training frequency. These two variables were consistently reported across all included studies and were explicitly identified in the introduction as potential sources of between-study variability. Due to inconsistencies in disease severity classification criteria across studies (e.g., different MMSE or CDR cutoffs) and incomplete reporting in some trials, subgroup analysis by disease stage was not performed.

#### 3.8.1 Subgroup analysis by intervention duration

The results of subgroup analyses stratified by intervention duration are presented in Figure 6.

**Figure 6:**
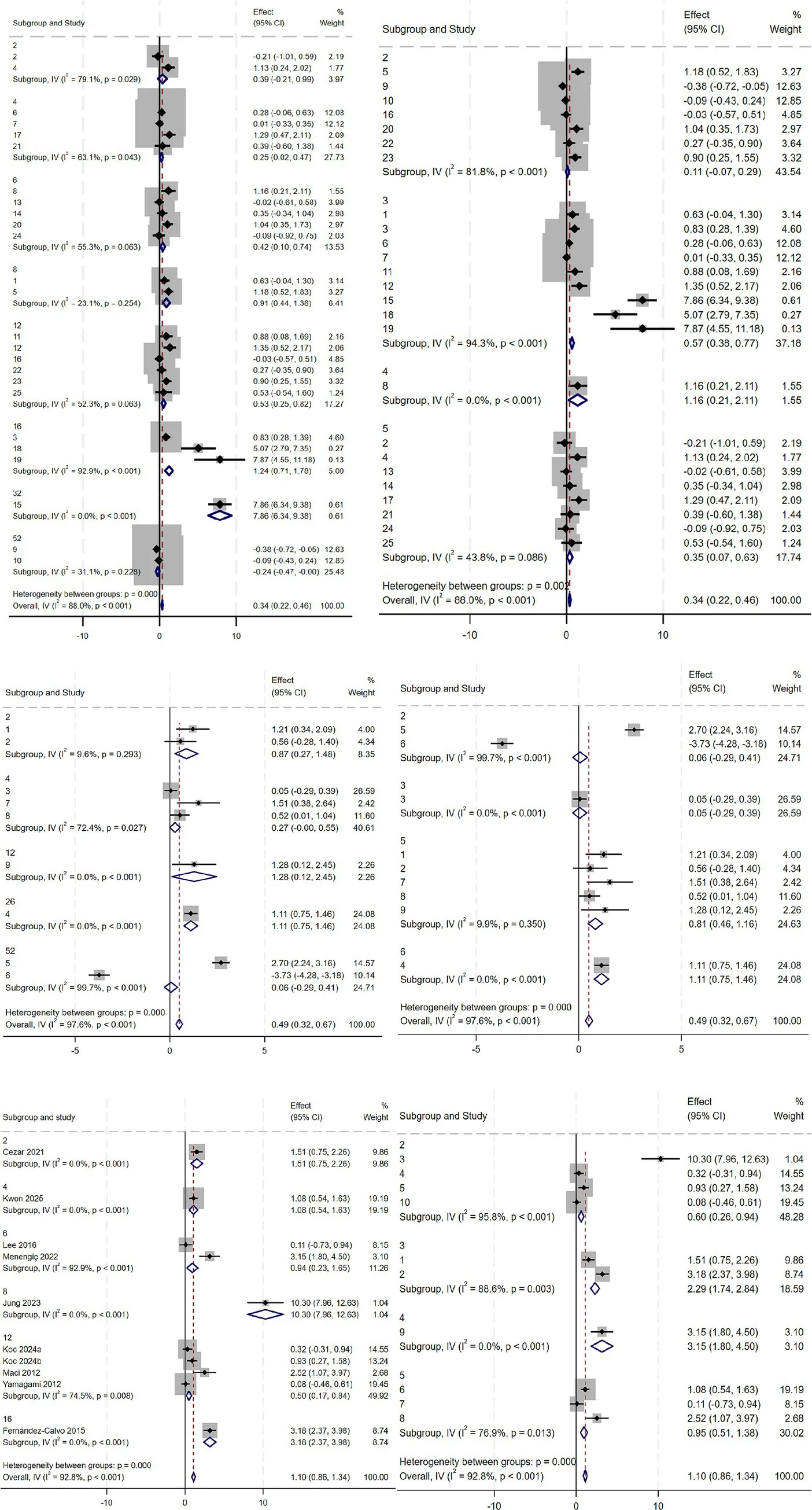
Subgroup analyses stratified by intervention duration and weekly training frequency for all outcomes.

For cognitive function, the effect sizes (SMD) for CET versus RC or other control conditions across different intervention durations were as follows: ≤4 weeks (SMD = 0.39, 95% CI: −0.21 to 0.99, *I*^2^ = 79.1%), 5–8 weeks (SMD = 0.25, 95% CI: 0.02 to 0.47, *I*^2^ = 63.1%), 9–16 weeks (SMD = 0.53, 95% CI: 0.25 to 0.82, *I*^2^ = 52.3%), and >16 weeks (SMD = −0.24, 95% CI: −0.47 to −0.00, *I*^2^ = 31.1%). The test for subgroup differences was statistically significant (*p* = 0.000), suggesting that intervention duration may moderate the cognitive effects of combined interventions. Notably, interventions lasting 5–8 weeks and 9–16 weeks showed significant cognitive benefits, whereas trials with very short (≤4 weeks) or very long (>16 weeks) durations did not demonstrate significant improvements, although the number of studies in the extreme duration groups was limited.

For executive function, subgroup analysis by intervention duration showed the following: ≤4 weeks (SMD = 0.87, 95% CI: 0.27 to 1.48, *I*^2^ = 9.6%), 5–8 weeks (SMD = 0.27, 95% CI: −0.00 to 0.55, *I*^2^ = 72.4%), 9–16 weeks (SMD = 1.28, 95% CI: 0.12 to 2.45, *I*^2^ = 0.0%), and >16 weeks (SMD = 0.06, 95% CI: −0.29 to 0.41, *I*^2^ = 99.7%). The subgroup difference was statistically significant (*p* = 0.000). Interventions lasting 9–16 weeks yielded the largest effect size for executive function, while the >16 weeks subgroup showed extremely high heterogeneity (*I*^2^ = 99.7%), indicating substantial variability among long-duration trials that warrants cautious interpretation.

For negative emotions, the subgroup results by intervention duration were: ≤4 weeks (SMD = 1.51, 95% CI: 0.75 to 2.26, *I*^2^ = 0.0%), 5–8 weeks (SMD = 0.94, 95% CI: 0.23 to 1.65, *I*^2^ = 92.9%), 9–16 weeks (SMD = 0.50, 95% CI: 0.17 to 0.84, *I*^2^ = 74.5%), and >16 weeks (SMD = 3.18, 95% CI: 2.37 to 3.98, *I*^2^ = 0.0%). The subgroup difference was statistically significant (*p* = 0.000). Notably, the longest duration (>16 weeks) yielded the largest effect size, suggesting that sustained interventions may be particularly beneficial for emotional outcomes, although only two trials were included in this subgroup.

#### 3.8.2 Subgroup analysis by weekly training frequency

The results of subgroup analyses stratified by weekly training frequency are presented in Figure 6.

For cognitive function, subgroup analysis by weekly frequency showed: ≤2 sessions/week (SMD = 0.11, 95% CI: −0.07 to 0.29, *I*^2^ = 81.8%), 3 sessions/week (SMD = 0.57, 95% CI: 0.38 to 0.77, *I*^2^ = 94.3%), 4 sessions/week (SMD = 1.16, 95% CI: 0.21 to 2.11, *I*^2^ = 0.0%), and 5 sessions/week (SMD = 0.35, 95% CI: 0.07 to 0.63, *I*^2^ = 43.8%). The subgroup difference was statistically significant (*p* = 0.002). Interventions delivered at 4 sessions per week showed the largest effect size, followed by 5 sessions per week, suggesting that higher weekly frequency may be associated with greater cognitive benefits, although the 4 sessions/week subgroup included only two studies.

For executive function, subgroup analysis by weekly frequency yielded: ≤2 sessions/week (SMD = 0.05, 95% CI: −0.29 to 0.39, *I*^2^ = 99.7%), 3 sessions/week (SMD = 0.56, 95% CI: −0.28 to 1.40, *I*^2^ = 0.0%), 4 sessions/week (SMD = 1.11, 95% CI: 0.75 to 1.46, *I*^2^ = 0.0%), and 5 sessions/week (SMD = 0.49, 95% CI: 0.32 to 0.67, *I*^2^ = 0.0%). The subgroup difference was statistically significant (*p* = 0.000). Similar to the cognitive function findings, interventions delivered at 4 sessions per week showed the largest effect size for executive function, although the ≤2 sessions/week subgroup exhibited extremely high heterogeneity, suggesting that the effect estimates in this subgroup are unreliable.

For negative emotions, subgroup analysis by weekly frequency showed: ≤2 sessions/week (SMD = 0.60, 95% CI: 0.26 to 0.94, *I*^2^ = 95.8%), 3 sessions/week (SMD = 2.29, 95% CI: 1.74 to 2.84, *I*^2^ = 88.6%), 4 sessions/week (SMD = 3.15, 95% CI: 1.80 to 4.50, *I*^2^ = 0.0%), and 5 sessions/week (SMD = 0.95, 95% CI: 0.51 to 1.38, *I*^2^ = 76.9%). The subgroup difference was statistically significant (*p* = 0.000). Interventions delivered at 4 sessions per week produced the largest effect size for improving negative emotions, followed by 3 sessions per week.

Overall, these subgroup analyses suggest that both intervention duration and weekly training frequency may contribute to the observed between-study heterogeneity, with medium-duration (5–16 weeks) and higher-frequency (≥3 sessions/week) interventions generally showing more favorable effect estimates. However, the substantial residual heterogeneity within several subgroups (*I*^2^ > 80%) and the limited number of studies in certain categories indicate that other unmeasured factors—including disease severity, intervention content, and outcome measurement tools—may also play important roles. These findings are exploratory in nature and should be interpreted with caution. Due to the limited number of studies, formal meta-regression was not performed, and the subgroup results should be considered hypothesis-generating rather than definitive.

### 3.9 Sensitivity analysis

To assess the robustness of the network meta-analysis results and to address the potential impact of study quality on the SUCRA rankings, we performed a sensitivity analysis by excluding studies rated as having a high risk of bias. Based on the Cochrane RoB 2.0 assessment (Table 2), six studies were identified as high risk and were excluded from this sensitivity analysis. The network meta-analysis was then re-run using the same frequentist random-effects model.

The results of the sensitivity analysis are presented in Figure 7. For cognitive function, after excluding high-risk studies, the pooled effect estimate remained significant (SMD = 0.34, 95% CI: 0.22 to 0.46, *I*^2^ = 88.0%), consistent with the main analysis. For executive function, the pooled effect estimate was also significant (SMD = 0.49, 95% CI: 0.32 to 0.67, *I*^2^ = 97.6%). For negative emotions, the pooled effect estimate remained significant (SMD = 1.10, 95% CI: 0.86 to 1.34, *I*^2^ = 92.8%).

**Figure 7:**
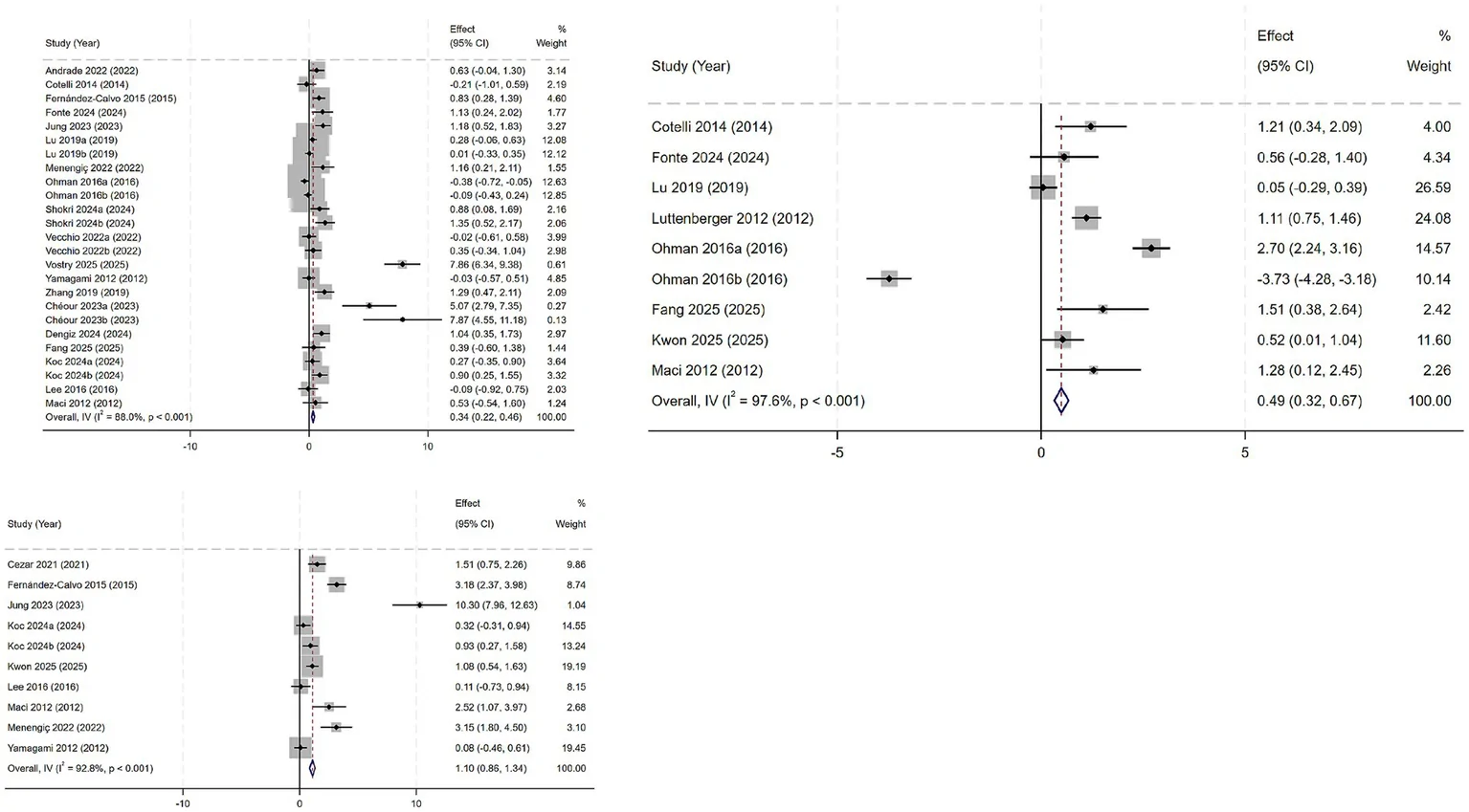
Sensitivity analysis forest plots for all outcomes after excluding high-risk-of-bias studies.

The SUCRA rankings derived from the sensitivity analysis were largely consistent with those from the main analysis. For cognitive function, CNIBS remained the highest-ranked intervention, followed by CET and CT. For executive function, CET maintained the highest SUCRA value. For negative emotions, CET continued to rank first, followed by PE. These findings suggest that the exclusion of high-risk-of-bias studies did not substantially alter the overall rankings, supporting the robustness of our primary results. However, the high heterogeneity persisted in the sensitivity analysis, consistent with the main analysis, indicating that variability across studies was not primarily driven by study quality alone. Due to the limited number of studies, we were unable to perform additional sensitivity analyses by excluding small-sample trials or switching to alternative effect size models (e.g., Hedges’ g or odds ratios for binary outcomes). These additional sensitivity analyses may be explored in future studies with larger evidence bases.

## 4 Discussion

This network meta-analysis systematically compared the effects of RC, CT, PE, CET, and CNIBS on cognitive function, executive function, and negative emotions in patients with AD. The results showed that the relative advantages of different interventions varied across outcomes: CNIBS ranked highest for cognitive function, followed by CET, whereas CET ranked highest for both executive function and negative emotions. These findings indicate that cognitive, executive, and emotional problems in patients with AD are multidimensional, and combined interventions may have more prominent application value in comprehensive rehabilitation management. The network meta-analysis findings should be considered hypothesis-generating rather than definitive. The counterintuitive finding for executive function - in which RC ranked second - highlights potential limitations in the current evidence network, underscoring the need for future standardized head-to-head RCTs.

### 4.1 Effects on cognitive function

The results of this study showed that CNIBS had the highest SUCRA ranking for improving cognitive function, followed by CET and CT. The main pooled effects showed that CET significantly improved cognitive function compared with RC, and CNIBS also showed a significant advantage over CT. This finding is consistent with previous systematic reviews on the effects of NIBS interventions on cognitive function in patients with AD and mild cognitive impairment (MCI). Previous studies have shown that NIBS can improve overall cognitive performance in patients with AD and MCI, with repetitive transcranial magnetic stimulation (rTMS) and transcranial direct current stimulation (tDCS) being the most commonly studied neuromodulation modalities (18, 26). CNIBS combines NIBS with CT, thereby jointly targeting cognition-related neural networks through cognitive task training and brain stimulation, which may explain its high ranking in this study.

CET ranked second for cognitive function and was significantly superior to RC, which is consistent with previous findings on combined cognitive and exercise interventions. A meta-analysis by Karssemeijer et al. (27) showed that CT combined with physical activity improved overall cognitive function in older adults with MCI or dementia. A systematic review by Muñoz-Perete et al. (28) also indicated that multicomponent interventions combining physical activity and cognitive stimulation have positive effects on cognitive health in older adults with cognitive impairment. Compared with PE alone, CET includes both physical activity and cognitive tasks, covering multiple cognitive processes such as attention, memory, response control, and task switching; therefore, it may be superior to single interventions for cognitive outcomes.

In this study, PE and RC ranked lower for cognitive function, which differs from some previous studies. A meta-analysis by Liu et al. (29) showed that PE had positive effects on cognitive performance in patients with AD, but differences existed across exercise forms, intervention periods, and outcome scales. In the present study, the number of PE studies included was limited, and substantial differences existed in exercise modalities and training doses across studies. Therefore, PE did not show a strong advantage in the network ranking. This result suggests that, in cognitive rehabilitation for AD, the cognitive benefits of PE alone may be lower than those of CET and CNIBS, which include cognitive stimulation components.

### 4.2 Effects on executive function

This study showed that CET had a markedly higher SUCRA value than other interventions for executive function. The pooled effects showed that CET was significantly superior not only to RC but also to PE. This result is consistent with the training characteristics of executive function. Executive function includes higher-order cognitive processes such as attentional allocation, working memory, inhibitory control, task switching, and planning ability. CET usually requires patients to simultaneously complete calculation, memory, language, response selection, or attentional switching tasks during exercise, and its training format is highly relevant to the core components of executive function.

Previous studies support the effects of combining exercise and CT on executive function. A meta-analysis by Zhu et al. (17) showed that physical activity can improve executive function, working memory, and cognitive flexibility in patients with AD. Karssemeijer et al. (27) also found that combined cognitive and physical activity interventions had positive effects on cognitive performance in patients with MCI or dementia. Compared with these studies, the present study further compared the relative effects of multiple interventions through network meta-analysis, showing that CET had the most prominent ranking advantage for executive function.

In this study, RC ranked second for executive function, whereas CT ranked lowest. This finding is not entirely consistent with traditional views in cognitive rehabilitation. Considering the network structure and pooled effect results of this study, the assessment tools used for executive function varied considerably across studies, and different scales measured different dimensions of executive function. In addition, scoring criteria may have differed among evaluators. These factors jointly increased the variability of executive function outcomes. Therefore, the relatively high ranking of RC and the low ranking of CT should be interpreted comprehensively in conjunction with effect sizes, confidence intervals, and evidence certainty.

### 4.3 Effects on negative emotions

The results of this study showed that CET ranked highest for improving negative emotions, followed by PE. The pooled effects showed that CET significantly improved negative emotions compared with RC, and CNIBS also showed a significant advantage over CT. This finding is consistent with previous studies on the effects of exercise interventions on neuropsychiatric symptoms in patients with AD. A systematic review by Mendonça et al. (30) indicated that PE can improve neuropsychiatric symptoms in older adults with AD. A meta-analysis by Dauwan et al. (31) also showed that PE, as an adjunctive non-pharmacological intervention, can improve quality of life, depressive symptoms, and overall functional status in patients with dementia.

CET ranked highest for negative emotions, indicating that the combination of cognitive tasks and exercise training has comprehensive advantages for emotional outcomes. Negative emotions in patients with AD are often interrelated with cognitive decline, reduced activity, decreased social participation, and limitations in daily functioning. CET provides physical activity, cognitive stimulation, and task engagement simultaneously, and the training process can enhance patients’ active participation and activity experience; therefore, it may be superior to RC and CT for emotional outcomes. The second-place ranking of PE further suggests that interventions containing an exercise component play an important role in improving emotional status in patients with AD.

CNIBS significantly improved negative emotions compared with CT, but its SUCRA ranking was lower than that of CET and PE. Previous studies have shown that NIBS has certain beneficial effects on cognitive and neuropsychiatric outcomes in patients with AD and MCI, but intervention effects are influenced by stimulation modality, stimulation site, and treatment course (18, 26). The findings of this study are consistent with the direction of the above evidence and further indicate that, for negative emotional outcomes, exercise-containing interventions such as CET and PE rank higher than CNIBS.

### 4.4 Clinical implications

Non-pharmacological interventions for patients with AD should be selected according to therapeutic goals. CNIBS and CET may be preferred for improving cognition; CET has the most obvious advantage for improving executive function; and CET and PE have higher value for alleviating negative emotions. In clinical practice, CET is suitable for patients with mild to moderate AD; CNIBS is suitable for patients whose primary goal is cognitive improvement and who are able to cooperate with treatment; PE is suitable for patients with reduced activity, low mood, or physical decline; and CT may serve as a basic intervention, although its effect alone is weaker than that of combined strategies. In the future, individualized and multicomponent rehabilitation strategies should be developed according to disease stage, exercise capacity, cognitive impairment profile, emotional status, and caregiving conditions.

It should also be noted that the included studies employed diverse outcome measurement tools—such as MMSE, ADAS-Cog, MoCA, and ACE-III for cognition; TMT-B, Stroop, Digit Span, and WCST for executive function; and GDS, NPI, HAMD, and HADS for negative emotions—which may capture slightly different aspects of the same construct. Although pooling these measures via SMD is methodologically justified, the heterogeneity in assessment instruments may affect the generalizability of the findings to specific clinical settings. When applying these results in practice, clinicians should be aware that the magnitude of improvement observed in a given study may partly reflect the characteristics of the measurement tool used. Future research should consider using standardized core outcome sets to facilitate cross-study comparisons and strengthen the evidence base for clinical decision-making.

Additionally, our cognitive function analyses were based on global screening instruments such as MMSE, MoCA, ADAS-Cog, and ACE-III. Although these tools are well-validated and widely used, they may not capture more nuanced domain-specific changes, such as memory or language. Future studies with larger sample sizes and more consistent use of domain-specific neuropsychological assessments are needed to confirm whether the observed treatment effects differ across specific cognitive domains.

We also acknowledge that our executive function analyses were based on a range of measures including TMT-B, Stroop, Digit Span, and WCST. Although these instruments are well-validated and widely used in AD research, they may emphasize different subdomains of executive function. The heterogeneity in executive function measures may therefore contribute to between-study variability. Future studies with more consistent use of standardized executive function batteries are needed to confirm the robustness of our findings across specific executive subdomains.

### 4.5 Limitations

This study has several limitations. First, considerable heterogeneity was observed across the included trials (*I*^2^ = 87–94%). To explore its sources, we conducted subgroup analyses stratified by intervention duration and weekly frequency. These analyses suggested that both factors may partially explain the variability, with medium-duration (5–16 weeks) and higher-frequency (≥3 sessions/week) interventions showing more favorable effects. However, substantial residual heterogeneity remained within several subgroups (*I*^2^ > 80%), and the limited number of studies precluded formal meta-regression; therefore, these subgroup analyses should be considered exploratory and hypothesis-generating. Formal subgroup analyses by CET subtype and NIBS modality were not performed due to insufficient study numbers. Second, the network estimates relied heavily on indirect evidence for some comparisons, particularly those involving PE and CNIBS; thus, the SUCRA rankings should be interpreted with caution. Third, blinding of participants and therapists was difficult to implement in non-pharmacological trials, which may have introduced performance bias. Fourth, the number of studies for certain intervention nodes was limited, and pooling diverse CET and CNIBS protocols may have obscured subtype-specific differences. Fifth, Egger’s tests revealed significant publication bias for cognitive function (bias = 4.79, *p* < 0.001) and negative emotions (bias = 7.84, *p* = 0.008), suggesting that small-study effects may have inflated effect estimates for these outcomes, whereas no such evidence was found for executive function (bias = 0.11, *p* = 0.986). Accordingly, the SUCRA rankings for cognitive function and negative emotions should be interpreted with particular caution, though we acknowledge that the limited number of studies may have reduced the power of Egger’s test to detect asymmetry. Sixth, the absence of long-term follow-up data limits our understanding of the durability of the observed effects. Seventh, although we performed a sensitivity analysis by excluding high-risk-of-bias studies, the limited number of included studies precluded additional sensitivity analyses, and the consistency tests may have been underpowered due to the small number of studies in some closed loops. Eighth, the assessment instruments for executive function varied considerably across the included studies, with the Trail Making Test Part B, Stroop Color-Word Test, and Wisconsin Card Sorting Test each capturing distinct subdomains of executive control—including cognitive flexibility, inhibitory control, and set-shifting, respectively. This conceptual heterogeneity may have contributed to the unexpected finding that remote collaboration (RC) ranked second for executive function, as different tests may be differentially sensitive to various intervention modalities. Future head-to-head trials employing standardized, multi-domain executive function batteries are warranted to validate these findings. Despite these limitations, this network meta-analysis provides a comprehensive comparison of five intervention strategies and offers clinically relevant evidence to guide intervention selection, though our findings require confirmation in future high-quality head-to-head RCTs.

In addition, although SMD pooling is methodologically justified for synthesizing different instruments measuring the same construct, the diversity of outcome measurement tools across studies remains a notable source of heterogeneity. Different scales may place varying emphasis on specific subdomains, and the lack of standardized equivalence across instruments means that the pooled effect sizes represent a general tendency rather than a scale-specific effect. However, due to the limited number of studies, we were unable to conduct subgroup analyses stratified by individual measurement tools, as this would have resulted in extremely small subgroups with insufficient statistical power to yield meaningful comparisons. Future studies with larger sample sizes and more consistent use of validated assessment tools are needed to confirm the generalizability of our findings across different measurement approaches.

## 5 Conclusion

This network meta-analysis compared the effects of five intervention strategies, namely RC, CT, PE, CET, and CNIBS, on cognitive function, executive function, and negative emotions in patients with AD. The results showed that combined interventions were generally superior to single interventions, supporting the rehabilitation value of multimodal combined strategies. CNIBS or CET may be preferred for improving overall cognition. For improving executive function or alleviating negative emotions, CET was the optimal choice, while PE alone also had unique value for emotional regulation.

Clinical decision-making should be individualized according to rehabilitation goals. When the goal is cognitive improvement, the two combined interventions should be prioritized. When the goal is improving executive function or emotional status, CET is the best option. For patients with reduced activity and low mood, PE alone may be considered. Overall, combined interventions centered on CT provide a better strategy for multidimensional symptom management in patients with AD. Future studies should optimize the parameters of combined intervention protocols and conduct long-term follow-up.

## Data Availability

The original contributions presented in the study are included in the article/Supplementary material, further inquiries can be directed to the corresponding authors.

## References

[1] Zheng Q Wang X. Alzheimer's disease: insights into pathology, molecular mechanisms, and therapy. Protein Cell. (2025) 16:83–120. doi: 10.1093/procel/pwae026, 38733347 PMC11786724

[2] Lopez-Lee C Torres ERS Carling G Gan L. Mechanisms of sex differences in Alzheimer's disease. Neuron. (2024) 112:1208–21. doi: 10.1016/j.neuron.2024.01.024, 38402606 PMC11076015

[3] Alzheimer’s Association. 2024 Alzheimer’s disease facts and figures. Alzheimers Dement. (2024) 20:3708–821. doi: 10.1002/alz.13809 PMC11095490 38689398

[4] Guarino A Favieri F Boncompagni I Agostini F Cantone M Casagrande M. Executive functions in Alzheimer disease: a systematic review. Front Aging Neurosci. (2019) 10:437. doi: 10.3389/fnagi.2018.00437, 30697157 PMC6341024

[5] Stefaniak O Dobrzyńska M Drzymała-Czyż S Przysławski J. Diet in the prevention of Alzheimer's disease: current knowledge and future research requirements. Nutrients. (2022) 14:4564. doi: 10.3390/nu14214564, 36364826 PMC9656789

[6] Zhao QF Tan L Wang HF Jiang T Tan MS Tan L . The prevalence of neuropsychiatric symptoms in Alzheimer's disease: systematic review and meta-analysis. J Affect Disord. (2016) 190:264–71. doi: 10.1016/j.jad.2015.09.069, 26540080

[7] Collins JD Henley SMD Suárez-González A. A systematic review of the prevalence of depression, anxiety, and apathy in frontotemporal dementia, atypical and young-onset Alzheimer's disease, and inherited dementia. Int Psychogeriatr. (2023) 35:457–76. doi: 10.1017/S1041610220001118, 32684177

[8] Marshall GA Rentz DM Frey MT Locascio JJ Johnson KA Sperling RA . Executive function and instrumental activities of daily living in mild cognitive impairment and Alzheimer's disease. Alzheimers Dement. (2011) 7:300–8. doi: 10.1016/j.jalz.2010.04.005, 21575871 PMC3096844

[9] Mohamed S Rosenheck R Lyketsos CG Schneider LS. Caregiver burden in Alzheimer disease: cross-sectional and longitudinal patient correlates. Am J Geriatr Psychiatry. (2010) 18:917–27. doi: 10.1097/JGP.0b013e3181d5745d, 20808108 PMC3972419

[10] da Silva AMP Falcão L Ribeiro Gonçalves O Virgilio Ribeiro F Machado Magalhães PL Lee Han M . Brexpiprazole for the treatment of agitation in older adults with Alzheimer's disease: a systematic review, Bayesian Meta-analysis, and Meta-regression. CNS Drugs. (2025) 39:1071–82. doi: 10.1007/s40263-025-01219-y, 40886227

[11] Livingston G Huntley J Liu KY Costafreda SG Selbæk G Alladi S . Dementia prevention, intervention, and care: 2024 report of the lancet standing commission. Lancet. (2024) 404:572–628. doi: 10.1016/S0140-6736(24)01296-0, 39096926

[12] National Institute for Health and Care Excellence (NICE). Dementia: Assessment, Management and Support for People Living with Dementia and Their Carers. London: National Institute for Health and Care Excellence. (2018). 30011160

[13] Risk Reduction of Cognitive Decline and Dementia: WHO Guidelines. Geneva: World Health Organization (2019). 31219687

[14] Woods B Rai HK Elliott E Aguirre E Orrell M Spector A. Cognitive stimulation to improve cognitive functioning in people with dementia. Cochrane Database Syst Rev. (2023) 1:CD005562. doi: 10.1002/14651858.CD005562.pub3, 39804128 PMC9891430

[15] Bahar-Fuchs A Martyr A Goh AM Sabates J Clare L. Cognitive training for people with mild to moderate dementia. Cochrane Database Syst Rev. (2019) 3:CD013069. doi: 10.1002/14651858.CD013069.pub2, 30909318 PMC6433473

[16] López-Ortiz S Valenzuela PL Seisdedos MM Morales JS Vega T Castillo-García A . Exercise interventions in Alzheimer's disease: a systematic review and meta-analysis of randomized controlled trials. Ageing Res Rev. (2021) 72:101479. doi: 10.1016/j.arr.2021.101479, 34601135

[17] Zhu L Li L Wang L Jin X Zhang H. Physical activity for executive function and activities of daily living in AD patients: a systematic review and Meta-analysis. Front Psychol. (2020) 11:560461. doi: 10.3389/fpsyg.2020.560461, 33343442 PMC7744293

[18] Chu CS Li CT Brunoni AR Yang FC Tseng PT Tu YK . Cognitive effects and acceptability of non-invasive brain stimulation on Alzheimer's disease and mild cognitive impairment: a component network meta-analysis. J Neurol Neurosurg Psychiatry. (2021) 92:195–203. doi: 10.1136/jnnp-2020-323870, 33115936 PMC7841477

[19] Chou YH Ton That V Sundman M. A systematic review and meta-analysis of rTMS effects on cognitive enhancement in mild cognitive impairment and Alzheimer’s disease. Neurobiol Aging. (2020) 86:1–10. doi: 10.1016/j.neurobiolaging.2019.08.020, 31783330 PMC6995441

[20] Law CK Lam FM Chung RC Pang MY. Physical exercise attenuates cognitive decline and reduces behavioural problems in people with mild cognitive impairment and dementia: a systematic review. J Physiother. (2020) 66:9–18. doi: 10.1016/j.jphys.2019.11.014, 31843427

[21] Vásquez-Carrasco E Jamett-Oliva P Quijada A Hernandez-Martinez J Branco BHM Carmine-Peña E . Effects of non-invasive brain stimulation combined with cognitive training on cognitive functions in older people with mild cognitive impairment: a systematic review with meta-analysis. Front Med. (2025) 12:1659208. doi: 10.3389/fmed.2025.1659208, 41140682 PMC12546134

[22] Salanti G. Indirect and mixed-treatment comparison, network, or multiple-treatments meta-analysis: many names, many benefits, many concerns for the next generation evidence synthesis tool. Res Synth Methods. (2012) 3:80–97. doi: 10.1002/jrsm.1037, 26062083

[23] Cumpston M Li T Page MJ Chandlerz J Welch VA Higgins JPT . Updated guidance for trusted systematic reviews: A new edition of the Cochrane Handbook for Systematic Reviews of Interventions. Cochrane Database of Systematic Reviews. (2019) 2019:ED000142. doi: 10.1002/14651858.ED000142 PMC10284251 31643080

[24] Huang X Zhao X Li B Cai Y Zhang S Wan Q . Comparative efficacy of various exercise interventions on cognitive function in patients with mild cognitive impairment or dementia: a systematic review and network meta-analysis. J Sport Health Sci. (2022) 11:212–23. doi: 10.1016/j.jshs.2021.05.003, 34004389 PMC9068743

[25] Dou J Zhang H Fu X Yang Y Gao X. Optimal dose and type of non-pharmacological treatments to improve cognitive function in people with Alzheimer's disease: a systematic review and network meta-analysis. Aging Ment Health. (2025) 29:228–37. doi: 10.1080/13607863.2024.2379427, 39028199

[26] Wu M Song W Wang X Teng L Li J Zhang J . Efficacy of non-invasive brain stimulation interventions on cognitive impairment: an umbrella review of meta-analyses of randomized controlled trials. J Neuroeng Rehabil. (2025) 22:22. doi: 10.1186/s12984-025-01566-3, 39910547 PMC11796046

[27] Karssemeijer EGA Aaronson JA Bossers WJ Smits T Olde Rikkert MGM Kessels RPC. Positive effects of combined cognitive and physical exercise training on cognitive function in older adults with mild cognitive impairment or dementia: a meta-analysis. Ageing Res Rev. (2017) 40:75–83. doi: 10.1016/j.arr.2017.09.003, 28912076

[28] Muñoz-Perete JM Carcelén-Fraile MDC Cano-Sánchez J Aibar-Almazán A Castellote-Caballero Y Mesas-Aróstegui MA . Combined physical-cognitive therapies for the health of older adults with mild cognitive impairment: a systematic review and Meta-analysis. Healthcare. (2025) 13:591. doi: 10.3390/healthcare13060591, 40150441 PMC11941773

[29] Liu W Zhang J Wang Y Li J Chang J Jia Q. Effect of physical exercise on cognitive function of Alzheimer's disease patients: a systematic review and Meta-analysis of randomized controlled trial. Front Psych. (2022) 13:927128. doi: 10.3389/fpsyt.2022.927128, 35782450 PMC9243422

[30] Mendonça DCB Fernandes DR Hernandez SS Soares FDG Figueiredo K Coelho FGM. Physical exercise is effective for neuropsychiatric symptoms in Alzheimer's disease: a systematic review. Arq Neuropsiquiatr. (2021) 79:447–56. doi: 10.1590/0004-282X-ANP-2020-0284, 34161531 PMC9394554

[31] Dauwan M Begemann MJH Slot MIE Lee EHM Scheltens P Sommer IEC. Physical exercise improves quality of life, depressive symptoms, and cognition across chronic brain disorders: a transdiagnostic systematic review and meta-analysis of randomized controlled trials. J Neurol. (2021) 268:1222–46. doi: 10.1007/s00415-019-09493-9, 31414194 PMC7990819

