# Supplementary material for: A meta-analysis of the effects of combined interventions centered on cognitive training on cognition, executive function, and negative emotions in patients with Alzheimer’s disease
Source: Front Neurol. 2026 Sep 15;17:1883995. doi: 10.3389/fneur.2026.1883995 (PMC13619425; doi:10.3389/fneur.2026.1883995)
Supplement: Supplementary file 1 [file Table_1.DOCX]

**Appendix 1** Basic features of the included studies

| Study | Country | Group | Sample size (M/F) | Age (mean ± SD) | Intervention duration | Intervention frequency |
| --- | --- | --- | --- | --- | --- | --- |
| Andrade 2022[1] | Brazil | CNIBS | 18 | 75.4 ± 4.7 | 8w | 30 min/session, 3 sessions/week |
|  |  | CT | 18 | 77.1 ± 5.2 | 8w | 30 min/session, 3 sessions/week |
| Cotelli 2014[2] | Italy | CNIBS | 12 (10/2) | 76.6 ± 4.6 | 2w | 25 min/session, 5 sessions/week |
|  |  | CT | 12 (9/3) | 74.7 ± 6.1 | 2w | 25 min/session, 5 sessions/week |
| Fonte 2024[3] | Italy | PE | 6 (4/2) | 88 ± 3 | 2w | 45 min/session, 5 sessions/week |
|  |  | CNIBS | 7 (4/3) | 78 ± 11 | 2w | 45 min/session, 5 sessions/week |
|  |  | CT | 4 (2/2) | 81 ± 5 | 2w | 45 min/session, 5 sessions/week |
| Lu 2019[4] | China (Hong Kong) | CNIBS | 69 (42/27) | 74.2 ± 6.7 | 4w | 45 min/session, 3 sessions/week |
|  |  | CT | 64 (36/28) | 74.5 ± 6.6 | 4w | 45 min/session, 3 sessions/week |
|  |  | CNIBS | 68 (30/38) | 73.4 ± 6.1 | 4w | 45 min/session, 3 sessions/week |
| Menengiç 2022[5] | Turkey | CET | 10 (7/3) | 77.7 ± 5.29 | 6w | Progressive (15-40 min), 4-5 days/week |
| Chéour 2023[6] | Tunisia | PE | 7 | M:74.19±4.27, F:71.46±3.36 | 16w | 60 min, 3 days/week |
|  |  | CT | 7 | M:74.19±4.27, F:71.46±3.36 | 16w | 60 min, 3 days/week |
|  |  | CET | 7 | M:74.19±4.27, F:71.46±3.36 | 16w | 60 min, 3 days/week |
| Shokri 2024[7] | Iran | PE | 13 | 67.5 ± 8.2 | 12w | 35-45 min/session, 3 days/week |
|  |  | CET | 13 |  | 12w | 35-45 min/session, 3 days/week |
| Vostry 2025[8] | Czech Republic | CET | 30 (28/2) | 69.2 | 8 months | 45-50 min/session, 3 days/week |
| Cezar 2021 (AD-HOMEX)[9] | Brazil | CET | 16 (14/2) | 79.75 ± 5.87 | 16 weeks | 60 min/session, 3 times/week |
| Cezar 2021 (March)[10] | Brazil | CET | 16 (14/2) | 79.7 ± 5.9 | 16 weeks | 60 min/session, 3 times/week |
| Dengiz 2024[11] | Turkey | CET | 18 (5/13) | 78.88 ± 2.08 | 6 weeks | Mangala: 2 times/week, 30 min; PT: 3 days/week |
|  |  | PE | 19 (12/7) | 79.63 ± 1.78 | 6 weeks | PT: 3 days/week |
| Ohman 2016[12] | Finland | PE | 70 (30/40) | 77.7 ± 5.4 | 52w (1 year) | 1 hour/session, 2 days/week |
|  |  | PE | 70 (25/45) | 78.3 ± 5.1 | 52w (1 year) | 1 hour active exercise/session, 2 days/week |
| Koc 2024[13] | Turkey | PE | 20 (11/9) | 78.9 ± 6.9 | 12w | 2 days/week, 60 min/session |
|  |  | CET | 20 (12/8) | 77.9 ± 6.6 | 12w | PE: 2 days/week, 60 min; CS: 3-5 days/week, 10 min |
| Fang 2025[14] | China | CNIBS | 8 (6/2) | 66.38 ± 7.71 | 4w | 5 days/week, 20 min/day |
|  |  | CT | 8 (5/3) | 66.25 ± 8.07 | 4w | 5 days/week, 20 min/day |
| Lee 2016[15] | Korea | CNIBS | 18 (10/8) | 72.1 ± 7.6 | 6w | 5 days/week |
| Vecchio 2022[16] | Italy | CNIBS | 30 (16/14) | 71.07 ± 6.84 | 6w | 5 days/week, 45-60 min/session |
|  |  | CT | 17 (7/10) | 72.24 ± 9.44 | 6w | 5 days/week, 45-60 min/session |
| Zhang 2019[17] | China | CNIBS | 15 (12/3) | 69.00 ± 8.19 | 4w | 5 times/week, 40 min/day |
|  |  | CT | 13 (10/3) | 68.54 ± 7.93 | 4w | 5 times/week, 40 min/day |
| Kwon 2025[18] | Korea | CET | 30 (14/16) | 72.43 ± 11.04 | 4w | 5 days/week (30 min + 50 min) |
|  |  | CT | 30 (16/14) | 71.53 ± 11.42 | 4w | 5 days/week (30 min + 50 min) |
| Maci 2012[19] | Italy | CET | 7 (4/3) | 75 ± 12.3 | 12w | 5 days/week, 3 hours/day |

[2] Andrade, S. M., Machado, D. G., Silva-Sauerc, L., Regis, C. T., Mendes, C. K. T. T., Araújo, J. S. S., Araújo, K. D. T., Costa, L. P., Queiroz, M. E. B. S., Leitão, M. M., & Fernández-Calvo, B. (2022). Effects of multisite anodal transcranial direct current stimulation combined with cognitive stimulation in patients with Alzheimer’s disease and its neurophysiological correlates: A double-blind randomized clinical trial. Neurophysiologie Clinique, 52, 117–127. <https://doi.org/10.1016/j.neucli.2022.02.003>
[3] Cotelli, M., Manenti, R., Brambilla, M., Petesi, M., Rosini, S., Ferrari, C., Zanetti, O., & Miniussi, C. (2014). Anodal tDCS during face-name associations memory training in Alzheimer’s patients. Frontiers in Aging Neuroscience, 6, 38. <https://doi.org/10.3389/fnagi.2014.00038>
[4] Fonte, C., Rotundo, G., Varalta, V., Filosa, A., Muti, E., Barletta, C., Evangelista, E., Venturelli, M., Picelli, A., & Smania, N. (2024). Combined Effect of tDCS and Motor or Cognitive Activity in Patients with Alzheimer's Disease: A Proof-of-Concept Pilot Study. Brain sciences, 14(11), 1099. <https://doi.org/10.3390/brainsci14111099>
[5] Lu, H., Chan, S. S. M., Chan, W. C., Lin, C., Cheng, C. P. W., & Lam, L. C. W. (2019). Randomized controlled trial of tDCS on cognition in 201 seniors with mild neurocognitive disorder. Annals of Clinical and Translational Neurology, 6(10), 1938–1948. <https://doi.org/10.1002/acn3.50823>
[6] Menengiç, K. N., Yeldan, İ., Çınar, N., & Şahiner, T. (2022). Effectiveness of motor-cognitive dual-task exercise via telerehabilitation in Alzheimer’s disease: An online pilot randomized controlled study. Clinical Neurology and Neurosurgery, 223, 107501. <https://doi.org/10.1016/j.clineuro.2022.107501>
[7] Chéour, S., Chéour, C., Gendreau, T., Bouazizi, M., Singh, K. P., Saeidi, A., Tao, D., Supriya, R., Bragazzi, N. L., Baker, J. S., & Chéour, F. (2023). Remediation of cognitive and motor functions in Tunisian elderly patients with mild Alzheimer's disease: implications of music therapy and/or physical rehabilitation. Frontiers in aging neuroscience, 15, 1216052. <https://doi.org/10.3389/fnagi.2023.1216052>
[8] Shokri, G., Mohammadian, F., Noroozian, M., Amani-Shalamzari, S., & Suzuki, K. (2024). Effects of remote combine exercise-music training on physical and cognitive performance in patients with Alzheimer’s disease: a randomized controlled trial. Frontiers in Aging Neuroscience, 15, 1283927. <https://doi.org/10.3389/fnagi.2023.1283927>
[9] Vostry, M., Chytry, V., Cmorej, P. C., Fleischmann, O., & Kubová, N. (2025). Effect of combined and intensive rehabilitation on cognitive function in patients with Alzheimer’s disease evaluated through a randomized controlled trial. Scientific Reports, 15(1), 8110. <https://doi.org/10.1038/s41598-025-93236-6>
[10] Cezar, N. O. C., Aprahamian, I., Ansai, J. H., Oliveira, M. P. B., Silva, D. C. P., Gomes, W. L., Barreiros, B. A., Langelli, T. C. O., & Andrade, L. P. (2021). Feasibility of reducing frailty components in older adults with Alzheimer’s dementia: a randomized controlled home-based exercise trial (AD-HOMEX). Experimental Gerontology, *150*, 111390. <https://doi.org/10.1016/j.exger.2021.111390>
[11] Cezar, N. O. C., Ansai, J. H., Oliveira, M. P. B., Silva, D. C. P., Gomes, W. L., Barreiros, B. A., Langelli, T. C. O., & Andrade, L. P. (2021). Feasibility of improving strength and functioning and decreasing the risk of falls in older adults with Alzheimer’s dementia: a randomized controlled home-based exercise trial. Archives of Gerontology and Geriatrics, 96, 104476. <https://doi.org/10.1016/j.archger.2021.104476>
[12] Dengiz, A., Cetisli-Korkmaz, N., & Kitis, A. (2025). Effect of the strategy game Mangala on cognitive function, anxiety, depression, and fine motor skills in individuals with Alzheimer’s disease: A randomized controlled trial. Applied Neuropsychology: Adult, 1–8. <https://doi.org/10.1080/23279095.2024.2448503>
[13] Öhman, H., Savikko, N., Strandberg, T. E., Kautiainen, H., Raivio, M. M., Laakkonen, M. L., Tilvis, R., & Pitkälä, K. H. (2016). Effects of Exercise on Cognition: The Finnish Alzheimer Disease Exercise Trial: A Randomized, Controlled Trial. Journal of the American Geriatrics Society, 64(4), 731–738. <https://doi.org/10.1111/jgs.14059>
[14] Akbuga Koc, E., Yazici-Mutlu, Ç., Cinar, N., & Sahiner, T. (2024). Comparison of the effect of online physical exercise and computerized cognitive stimulation in patients with Alzheimer's disease during the Covid-19 pandemic. Complementary therapies in clinical practice, 57, 101881. <https://doi.org/10.1016/j.ctcp.2024.101881>
[15] Fang, J., Shi, Y., Ba, L., Zhang, M., Li, M., Zheng, N., Qin, Y., & Zhu, W. (2025). Gray matter and white matter functional connectivity changes induced by rTMS concurrent with cognitive training in Alzheimer's disease. Brain research bulletin, 228, 111418. <https://doi.org/10.1016/j.brainresbull.2025.111418>
[16] Lee, J., Choi, B. H., Oh, E., Sohn, E. H., & Lee, A. Y. (2016). Treatment of Alzheimer's Disease with Repetitive Transcranial Magnetic Stimulation Combined with Cognitive Training: A Prospective, Randomized, Double-Blind, Placebo-Controlled Study. Journal of clinical neurology (Seoul, Korea), 12(1), 57–64. <https://doi.org/10.3988/jcn.2016.12.1.57>
[17] Vecchio, F., Quaranta, D., Miraglia, F., Pappalettera, C., Di Iorio, R., L'Abbate, F., Cotelli, M., Marra, C., & Rossini, P. M. (2022). Neuronavigated Magnetic Stimulation combined with cognitive training for Alzheimer's patients: an EEG graph study. GeroScience, 44(1), 159–172. <https://doi.org/10.1007/s11357-021-00508-w>
[18] Zhang, F., Qin, Y., Xie, L., Zheng, C., Huang, X., & Zhang, M. (2019). High-frequency repetitive transcranial magnetic stimulation combined with cognitive training improves cognitive function and cortical metabolic ratios in Alzheimer's disease. Journal of neural transmission (Vienna, Austria : 1996), 126(8), 1081–1094. <https://doi.org/10.1007/s00702-019-02022-y>
[19] Kwon, D., & Kim, D. (2025). Effects of cognitive stimulation group programs in patients with mild Alzheimer's disease. The British journal of occupational therapy, 88(8), 467–478. <https://doi.org/10.1177/03080226251314978>
[20] Maci, T., Pira, F. L., Quattrocchi, G., Nuovo, S. D., Perciavalle, V., & Zappia, M. (2012). Physical and cognitive stimulation in Alzheimer Disease. the GAIA Project: a pilot study. American journal of Alzheimer's disease and other dementias, 27(2), 107–113. https://doi.org/10.1177/1533317512440493

**Appendix 2** Risk of bias assessment graph of the included studies.

**Appendix 3** Forest plots of pairwise comparisons among different rehabilitation interventions for improving cognitive function, executive function, and negative emotion
